# Molecular detection of colistin resistance genes (*mcr*-*1* to *mcr*-*5*) in human vaginal swabs

**DOI:** 10.1186/s13104-018-3255-3

**Published:** 2018-02-20

**Authors:** Jilei Zhang, Li Chen, Jiawei Wang, Patrick Butaye, Ke Huang, Haixiang Qiu, Xiaomei Zhang, Weijuan Gong, Chengming Wang

**Affiliations:** 1 Yangzhou University College of Veterinary Medicine, Yangzhou, 225009 China; 2 Department of Biosciences, Ross University School of Veterinary Medicine, P.O. Box 334 Basseterre, Saint Kitts and Nevis; 3 Department of Pathology, Bacteriology and Poultry Diseases, Faculty of Veterinary Medicine, Ghent University, Ghent, Belgium; 4 Subei People’s Hospital, Yangzhou, Jiangsu China; 5 Yangzhou University College of Medicine, Yangzhou, Jiangsu 225009 People’s Republic of China; 6 College of Veterinary Medicine, Auburn University, Auburn, AL USA

**Keywords:** Colistin resistance, *mcr* genes, qPCR, Human vaginal swabs, Phylogenetic comparison

## Abstract

**Objective:**

Colistin resistance has emerged worldwide and has been threatening the efficacy of one of the last-resort antimicrobials used for treatment of multidrug resistant Gram-negative bacteria. While five colistin resistance genes (*mcr*-*1*, *mcr*-*2*, *mcr*-*3*, *mcr*-*4* and *mcr*-*5*) have been described, few data are available on the prevalence of *mcr*-genes other than *mcr*-*1* in human samples.

**Results:**

In this study, the presence of five currently described colistin resistance genes (*mcr 1*–*5*) in vaginal swabs of women undergoing infertility evaluation was reported. Most samples were found to be positive for the *mcr*-*4* (12.7%), followed by two for the *mcr*-*2* (1.5%), two for the *mcr*-*3* (1.5%), one for the *mcr*-*1* (0.7%), and one for the *mcr*-*5* (0.7%). Phylogenetic comparison demonstrated identical (*mcr*-*1*, *mcr*-*2*, *mcr*-*3*, *mcr*-*5*) or similar (*mcr*-*4*) nucleotide sequences of human samples and those of animal origins from the same city, suggesting the potential transmission of *mcr* genes from animals to humans. This is the first detection of *mcr*-*2*, *mcr*-*4* and *mcr*-*5* genes in human samples, and warrants further research to determine the spread of the *mcr* genes and elucidate the full epidemiology of colistin resistance genes in humans.

## Introduction

Antimicrobial resistance is one of the most serious global threats to human health, especially the multiple drug resistant-pathogens of ESKAPE group (*Enterococcus faecium*, *Staphylococcus aureus*, *Klebsiella pneumoniae*, *Acinetobacter baumannii*, *Pseudomonas aeruginosa* and *Enterobacter* spp.). The reintroduction of the older and less user-friendly antibiotics such as colistin is an option for treatment of the infections with bacteria of ESKAPE group. However, the efficiency of colistin treatment is compromised by the presence of an increasing number of mobile colistin resistance (*mcr*) genes. Recent findings indicate a low prevalence of *mcr*-*1* in *Enterobacteriaceae* from inpatients and healthy volunteers (≤ 1%) [1]. Up to the preparation of this manuscript, there are five colistin resistance genes described (*mcr*-*1*, *mcr*-*2*, *mcr*-*3*, *mcr*-*4* and *mcr*-*5*) [2–6]. However, few data are available on the prevalence of *mcr*-genes other than *mcr*-*1* in human samples [1, 2, 7–9]. In this study, PCRs were used to determine the presence of five *mcr* genes in human vaginal swabs, and phylogenetic comparison was performed the nucleotide similarity of the *mcr* genes from human and animals in the same city.

## Main text

### Materials and methods

This study received permission from the patients and was approved by the Institutional Review Board of Subei People’s Hospital. In 2016, vaginal swabs were collected from 134 women attending Subei People’s Hospital of Yangzhou in China for first or second infertility evaluation. Previous study demonstrated that none of vaginal swabs were positive for *Neisseria gonorrhoeae* and *Treponema pallidum*, but 18.8% of these swabs were positive for *Chlamydia trachomatis* and 17.3% of the swabs were positive for *Mycoplasma* species by PCRs [10]. All swabs were positive for tetracycline resistance gene *tet*(M) which is the most effective antibiotic for bacterial sexually transmitted infections [10].

Collection and DNA extraction of the human vaginal swabs were performed as described before [10]. Nucleotides of *mcr*-*1*, *mcr*-*2* and *mcr*-*3* genes in the samples were amplified with primers described before [11]. Meanwhile, using the Clustal Multiple Alignment Algorithm, we developed and validated a *mcr*-*4*-PCR (forward primer: 5′-AATTGTCGTGGGAAAAGCCGC-3′; reverse primer: 5′-CTGCTGACTGGGCTATTACCGTCAT-3′; amplicon size 1062 bp), and a *mcr*-*5*-PCR (forward primer: 5′-GTGAAACAGGTGATCGTGACTTACCG-3′; reverse primer: 5′-CGTGCTTTACACCGATCATGTGCT-3′; amplicon size 271 bp) in this study. The specificity of the primers for *mcr*-*4* and *mcr*-*5* PCRs were verified by BLASTN and DNA sequencing of the obtained PCR products. The sensitivity of the *mcr*-*4*-PCR and *mcr*-*5*-PCR was determined by amplifying dilutions of synthesized plasmids containing portions of the target *mcr*-*4* and *mcr*-*5* which were linearized with *Sac I* (Takara Biothechnology, Dalian, China). The quantitative standards were quantified using the PicoGreen DNA fluorescence assay (Molecular Probes, Eugene, OR, USA) for preparation of standards (10^4^, 10^3^, 10^2^, 10^1^, and 10^0^ copies/reaction). The PCR products were confirmed by gel electrophoresis, and DNA sequencing with both PCR primers (BGI, Beijing, China) after purification with QIAquick Gel Extraction Kit (Qiagen, Valencia, CA, USA).

In this study, every 24th samples tested consisted of diethylpyrocarbonate-treated ddH_2_O, serving as a negative extraction control to confirm the absence of contamination between samples during DNA extraction and carry-over contamination. Additionally, control swabs from pipettes, experimental benches and centrifuges were frequently processed for five *mcr*-PCRs to verify that no false amplification occurred during this study resulting from carry-over contamination.

All reported human *mcr* sequences, representative *mcr* sequences from animals, and the *mcr* sequences from animals at Yangzhou were aligned with the obtained *mcr* sequences in this study. Based on these alignments, phylogenetic trees were constructed by the neighbor-joining method using the Kimura 2-parameter model with MEGA 6.0. The Bootstrap values were calculated using 500 replicates. The BLASTN was performed to determine new *mcr* variants by comparing the *mcr* sequences from this study and those available in GenBank.

### Results and discussion

The *mcr* PCRs described before (*mcr*-*1*, *mcr*-*2* and *mcr*-*3*) and established in this study (*mcr*-*4* and mcr-5) were confirmed to specifically amplify the intended *mcr* gene, but not other *mcr* genes. The detection limit for *mcr*-*4* PCR and *mcr*-*5* PCR were determined to be 10 copies per 20 µL reaction.

Of 134 human swab samples, 22 of the vaginal swab (16.4%) were positive for at least one *mcr* gene. Most samples were found to be positive for the *mcr*-*4* gene (12.7%), followed by two for the *mcr*-*2* gene (1.5%), two for the *mcr*-*3* gene (1.5%), one for the *mcr*-*1* gene (0.7%), and one for the *mcr*-*5* gene (0.7%) (Table 1). The single swab sample positive for *mcr*-*5* was also positive for *mcr*-*4*. This is a much higher prevalence of *mcr* genes than in every other human sample taken to date, but a similar prevalence for the *mcr*-*1* gene previously reported in bacterial isolates from humans in China (Table 1) [1, 2, 12].

**Table 1:** The *mcr* genes in human vaginal swabs identified in this study

*mcr*-*1*	*mcr*-*2*	*mcr*-*3*	*mcr*-*4*	*mcr*-*5*
1				
	2			
		2		
			17	1

Furthermore, the *mcr*-*4* gene was also reported in *Salmonella* strains isolated from human of Italy [13]. While direct PCR testing is ideal for the rapid estimation of risk and risk analysis, it does not readily enable investigations of movement of resistance between bacteria of the same or different species. As we did not base the study on the isolation of the bacteria, we are not sure which bacteria are carrying these genes, or on what mobile genetic elements these genes are carried. It is striking that the *mcr*-*4* gene was so prevalent and this urges for more studies into the importance of this gene in colistin resistance.

**Fig. 1:**
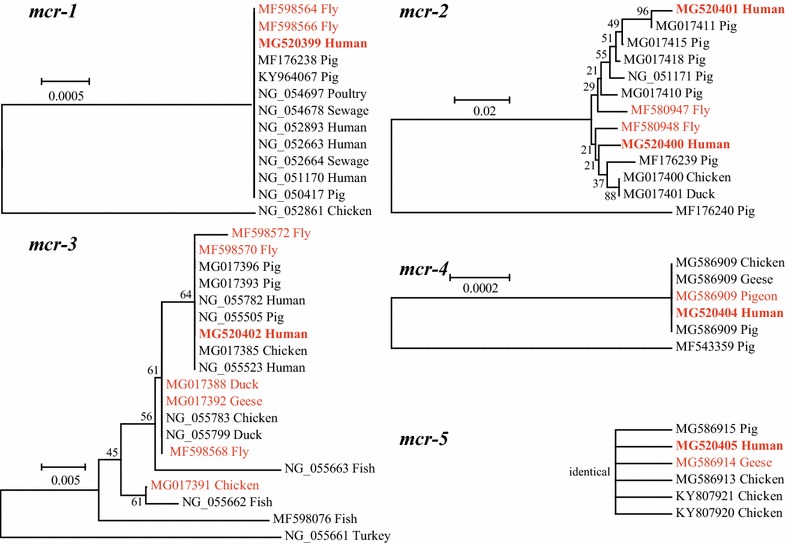
Phylogenetic tree of sequences in five *mcr* genes. The nucleotide sequences of colistin resistance genes (*mcr*-*1*, 342 bp; *mcr*-*2*, 282 bp; *mcr*-*3*, 267 bp; *mcr*-*4*, 1062 bp; *mcr*-*5*, 197 bp) in human beings obtained this study (in bold font) were compared with those of representative *mcr* variants obtained from NCBI. The sequences from animals in Yangzhou, the same city for human samples, are shown with red font. The evolutionary history was inferred using the Neighbor-Joining method. The tree is drawn to scale, with branch lengths in the same units as those of the evolutionary distances used to infer the phylogenetic tree

In this study, we identified two new *mcr*-*2* variants (MG520400, MG520401) and two new *mcr*-*4* variants (MG520403, MG520404), while nucleotide sequences of *mcr*-*1* (MG520399), *mcr*-*3* (MG520402) and *mcr*-*5* (MG520405) were identical to the original description of those genes (*mcr*-*1*: NG_054417; *mcr*-*3*: NG_055505; *mcr*-*5*: KY807920) (Fig. 1) [2, 4, 6]. Phylogenetic analysis demonstrates the identical (*mcr*-*1*, *mcr*-*2*, *mcr*-*3*, *mcr*-*5*) or similar (*mcr*-*4*) nucleotide sequences of human samples from Yangzhou in this study and those of animal origins in the same city (Fig. 1). This suggests the potential transmission of *mcr*-positive bacteria and/or *mcr* genes from animals to human beings. Therefore, it is more important for the government and companies to keep the food-productive animals from contaminating with *mcr* genes to ensure the food safety for human beings.

In conclusion, we found all the current described colistin resistance genes in vaginal swabs with a surprisingly high prevalence of *mcr*-*4*. This is the first detection of *mcr*-*2*, *mcr*-*4* and *mcr*-*5* genes in human samples. Further studies on other samples and including cultivation of the *mcr*-carrying bacteria should be performed to determine the exact spread of these genes in bacteria from humans and elucidate the full epidemiology of colistin resistance genes in humans.

## Limitations

The main limitation of this study is that the detection of *mcr* genes in vaginal swabs was solely based on quantitative PCRs, DNA sequencing and phylogenetic comparison. The background information such as prior treatment history of the patients was not available to this investigation. In future investigations, it would be useful to isolate *mcr* positive bacteria, and determine the species of the *mcr*-carrying bacteria, and whether the *mcr* genes are carried by plasmids or on the chromosome.

## Abbreviations

*mcr*: mobile colistin resistance
ESKAPE group: *Enterococcus faecium*, *Staphylococcus aureus*, *Klebsiella pneumoniae*, *Acinetobacter baumannii*, *Pseudomonas aeruginosa* and *Enterobacter* spp.
